# Supplementary material for: Are carbon emissions trading and green financial instruments synergistic? -Comprehensive quantitative research based on content analysis
Source: PLoS One. 2024 Mar 7;19(3):e0298601. doi: 10.1371/journal.pone.0298601 (PMC10919601; doi:10.1371/journal.pone.0298601)
Supplement: S1 Data — (ZIP) [file pone.0298601.s001.zip › Data/地级市Z_F工作报告.zip!/╜¡╬≈/╝¬░▓╩╨/└┤╫╘╣1⁄2╓┌║┼í╛┬φ┐╦╩2╛▌═°í┐_2011.docx]

各位代表：

　　现在，我代表市人民政府向大会报告工作，请予审议，并请各位市政协委员和列席会议的同志提出意见。

　　一、2010年工作和“十一五”经济社会发展的回顾

　　2010年是极不平凡的一年。我们在省委、省政府和市委的正确领导下，以科学发展观为指导，齐心协力，沉着应对，奋力拼搏，扎实工作，深入实施“主攻项目、决战两区”战略，全力以赴抓发展、促改革、惠民生，圆满完成市二届人大六次会议提出的目标任务。

　　——经济实力明显提升。全年完成生产总值665亿元，增长14%；财政总收入88.62亿元，增长37.7%，地方财政收入57亿元，增长43.5%，税收占财政收入比重上升到81.4%；规模工业增加值250.43亿元，增长27.3%，主营业务收入突破千亿元大关，工业经济效益综合指数提高51.9个百分点；社会消费品零售总额202亿元，增长18%；外贸出口突破10亿美元，增长125.4%。县域经济加快发展，8个县（区）财政总收入过5亿元，安福、泰和县过8亿元，吉安县突破10亿元，全市进入提速发展的快车道。

　　——发展后劲明显增强。全市完成固定资产投资766亿元，增长30%。市重点调度的330 个重大项目274个开工建设，石吉高速、吉泰走廊快速通道建成通车，峡江水利枢纽、衡茶吉铁路、吉莲高速等重点工程加快建设；全市三次产业结构调整为21:50.5:28.5，投资结构继续优化，亿元以上竣工、在建工业项目240个，完成工业投资300亿元，增长66%，工业对经济的贡献率达62%。蓄积了新的发展能量。

　　——城乡面貌明显变化。围绕打造“山水庐陵、生态吉安”，各地以文化为灵魂、绿化为脉络，在坚持品位中拉开城镇框架，完善城镇功能。一批基础性、功能性、民生性、生态性城建项目竞相开工落成。全市实施城建重点项目415个，完成投资228.13亿元，庐陵文化生态园、吉安县天祥公园、青原区东井冈公园、安福县和遂川县文化公园等一批园林精品尽显生态宜居品质。重点小城镇和新农村建设顺利推进，城乡面貌正在发生深刻变化。

　　——群众生活明显改善。全年用于民生事业的一般预算支出84.15亿元，积极解决教育、卫生、就业、社会保障等事关人民群众切实利益问题，省政府下达的93项民生指标全面或超额完成。城镇居民人均可支配收入15364元，增长9%；农村居民人均纯收入5418元，增长8%。年末城乡居民储蓄存款余额574.3亿元，比年初增长11.9%。全市贫困人口由18.5万人减少到16.6万人。

　　一年来，我们突出抓了以下工作：

　　（一）集聚产业决战园区，工业发展进一步提速。全市工业园区基础设施建设再投入37亿元，建成标准厂房75万平方米，园区承载功能日益完善。井开区晋升国家级经济技术开发区，列为国家生物源农药高新技术产业化基地。继井开区之后，泰和、吉安县工业园跻身百亿元园区，井开区、吉州区工业园跻身全省生态园区。吉泰走廊加速集聚，区域规模工业企业达371家，其中电子信息产业完成主营业务收入126亿元，占全市行业比重达84%，被列为全省风能核能及节能技术和电子信息产业基地、全省LED战略性新兴产业配套基地。以工业园区为载体，主攻亿元以上重大工业项目，三江超纤二期、优特利锂动力电池、航盛车载电子、正嘉谷物、嘉泰电子、永丰400万吨旋窑水泥等一大批重大项目先后竣工投产。全市主营业务收入上亿元企业达240户，“6＋1”支柱产业完成主营业务收入850亿元，占全市规模工业的85%；新材料、LED照明、生物新医药等战略性新兴产业加快发展，主营业务收入增长83%，高新技术产业主营收入增幅列全省第二。工业主导型经济增长格局已经形成。

　　（二）统筹城乡决战城区，新型城镇化进程进一步加快。出台一系列推进新型城镇化、加快示范镇建设的政策措施。全市城市基础设施和公建投资87.42亿元，一批城市主次干道、学校、医院、排污、垃圾处理等基础项目先后建成，城镇综合承载力明显增强。中心城区市文化艺术中心、城南和滨江路网等55项工程竣工；首次举办城建项目招商会，多元投资城市建设格局正在形成。城市管理水平不断提升，以传承庐陵古韵、保护绿色生态、提高老百姓幸福指数为导向，通过“五位一体”的老城区改造，中心城区一批干道街景改造完工，一批老住宅区和小街小巷修缮完成；调整城管环卫体制，逐步实现由突击管理向长效管理转变；出台物业管理办法，城市物业管理有所加强。吉泰城市群规划全面启动，公交、电信一体化基本实现。在乡镇规范化建设基础上，新一轮示范镇建设全面展开。全市城镇化率达40.85%，比上年提高2.01个百分点，城镇建成区面积增加25.7平方公里，新增城镇人口13.5万人。

　　（三）抗洪救灾夺取胜利，“三农”工作进一步加强。努力克服特大洪涝灾害带来的不利影响，积极发展现代农业，促进农业增效、农民增收。面对去年6月历史罕见的特大洪涝灾害，我们见事早、决策明、行动快，动员一切力量全力以赴投入抗洪抢险救灾，展现出众志成城、英勇顽强、奋力抗争、务求必胜的精神风貌，转移安置被困群众27.57万人次，发放救灾救济资金3109万元，确保了堤坝安全、确保了水库安全、确保了人民群众生命财产安全，得到省委、省政府和全市人民的充分肯定。灾后，全市迅速组织万名干部进村入户帮扶灾区重建，恢复生产。农业在大灾之年喜获丰收。全年粮食播种980万亩，总产373.6万吨，实现连续七年增产；畜禽、蔬菜、水产、井冈蜜柚、高产油茶等产业迅速发展，全市蔬菜面积135万亩，生猪出栏380万头，肉牛出栏40万头，特种水产品17万吨；积极探索土地流转，新增高产油茶6万亩、井冈蜜柚１万亩。全市新增市级龙头企业21家、省级龙头企业6家，总量达223家；新增农民专业合作社451家，总量达1226家，产业化经营水平提高。井冈山农业科技园升格为国家级农业科技园，“狗牯脑”茶叶获上海世博会绿茶类金奖。新农村建设扎实推进。累计投入1.4亿元，又有833个村完成“三清六改”，井冈山市获全省新农村建设五年标兵县（市）；716公里国省道公路“迎国检”任务全面完成，农村改渡建桥22座，改造县乡公路300公里，建成通村水泥路1339公里。农业基础设施建设掀起高潮。实施水利建设大会战，加固堤防82.6公里，修复水毁工程3468处，除险加固水库113座，改造中低产田13万亩，建设高标准农田7万亩。扶贫和移民工作取得新成效，完成库区、深山区移民搬迁13381人，峡江水利枢纽移民安置顺利启动。

　　（四）突出商贸市场建设，现代服务业进一步壮大。努力完善商贸物流体系，做大市场规模，扩大城乡消费，消费拉动经济增长5.1%。科学规划市中心城区电子数码城、生产生活资料、农副产品、高端商城、汽车综合市场等十大专业市场，加强中心城区菜市场建设和管理，竹笋巷、康居农贸市场竣工投用；新世界广场、吉安农副产品物流中心、天虹商城开工建设，全市建成各类商贸市场312个，货运配载中心98家；首届春节商品交易会取得成效，积极组织家电下乡活动，全市销售总量28.06万台（件），新改造建成“万村千乡市场工程”农家店330家。旅游再现红火。成功举办首届中国井冈山国际杜鹃花节、井冈山红色旅游高峰论坛。梨坪国际会议中心建设稳步推进，武功山景区温泉度假村基本建成，渼陂古村成功创建4A景区，新增青原山风景区等8家3A级景区。井冈山机场旅客吞吐量突破18万人次，全市接待游客1414.6万人次，实现旅游总收入90.1亿元。

　　（五）深入推进改革开放，发展活力进一步增强。开放领域不断扩大。全年引进内资185.94亿元，增长43.27%，实际利用外资4.4亿美元，增长27.6%；日本住友电装、胜美达电机、德国量一光电、中信华电子、宝成鞋业等项目纷纷落户；拓展城市建设、现代农业、金融商贸领域招商，全市引进5000万元以上一产、三产项目49个，合同引资总额168.6亿元。出口结构逐步优化。以电子信息为主的机电产品连续20个月成为我市第一大出口商品，增长130%，出口最终目的国（地区）达150个，新增61个，出口增幅列全省第三。各项改革排难而进。完成非工口七大系统144户国企改革，市公交公司、市供水公司、部分县粮食企业改制分别与江西长运公司、中国水务集团、江粮集团等战略投资者合作经营；市、县政府机构改革基本完成，深化乡镇机构改革；财政体制改革不断深化，执收执罚部门收支脱钩改革全面推开，事业单位绩效工资改革、机关公务员及参公单位人员津补贴提标顺利实现；网上审批、电子监察应用系统正式运行，行政审批再精简。金融工作又获佳绩。吉州、青原农信社重组筹建吉安农村商业银行已完成募股，九江银行在泰和、安福设立县级支行，赣州银行落户开业，吉安县、井冈山市分别引进浙江稠州、九江村镇银行，吉州银丰小额贷款公司顺利开业，天人生态股份有望在深圳交易所挂牌上市。全市银行金融机构各项贷款余额375亿元，比年初增加63亿元，增长20.2%。

　　（六）注重环境生态建设，绿色生态优势进一步凸显。策应鄱阳湖生态经济区建设，出台促进低碳与绿色生态发展的政策措施，引领全市积极调整优化产业结构，努力打造“低碳吉安”。 “一大四小”工程建设富有成效，共投入资金4.51亿元，完成造林绿化85.8万亩，我市和6个县（市）评为全省先进，全市森林覆盖率达66.5%；一批高标准的通道和城市绿化景观先后建成，8个乡镇被命名为全国生态乡镇。中心城区和县城全面启动森林城市创建，对峡江、石虎塘水利枢纽淹没区名木古树实施抢救性移栽。环境监管与保护力度加大，对造纸、冶金等重点行业和赣江、禾河等重要水系两岸污染进行了综合整治，立案查处环境违法企业106家，节能减排任务全面完成，生态环境有效保护和改善，环境质量全省一流。

　　（七）切实保障和改善民生，和谐平安局面进一步巩固。坚持富民为先，民生为重。社会就业稳步向好，安排就业再就业专项资金1.6亿元，购买城乡基层公共服务公益性岗位6058个，城镇新增就业12.5万人，新增转移农村劳动力16.8万人。社会保障体系逐步完善，城镇职工基本养老金人均提标146元，高于全省平均标准；失地农民生活保障长效机制初步建立；城乡居民基本医疗保险全员覆盖，试点农村养老保险；城乡低保对象月人均补差提标；针对物价上涨较快，及时发放困难群众生活补贴。住房保障体系初步建立，全市开工建设廉租住房5524套，经济适用住房6.8万平方米，完成棚户区改造113万平方米，为2468户倒房灾民发放建房补助资金2246万元，为6563户农村困难群众维修、新建住房。安全生产、社会治安保持平稳态势，全市公众安全感居全省第四，人民群众幸福感和安全感进一步提升。

　　（八）坚持统筹协调发展，社会事业进一步完善。科技进步，全市获批国家和省级科技项目48项，省优秀科技新产品22项，杰克机床获中国机械工业联合会科技进步一等奖，大圣塑料光纤获省科技进步一等奖。投资教育力度加大，落实了56.5万名义务教育阶段学生“两免一补”政策，资助各类贫困学生8.8万名；吉安新白鹭洲、特殊教育等四所学校向新城区布点启动建设，成功引进言爱基金建设新校；启动市教育园区建设；提高非义务教育阶段学校教师津补贴标准。文体卫生事业加快发展，农村文化“三项活动”深入开展，农家书屋建设进一步推进；永新小鼓《宝朵招工》获全国第十五届“群星奖”；天腾科技创意文化产业园落户吉州，填补我市空白；首部《吉安地区志》出版；群众体育运动蓬勃开展，竞技体育实力有所增强；“光明·微笑”工程和儿童“两病”免费救治工作继续扎实推进，72所中心卫生院全部实施国家基本药物制度；食品药品安全保障能力得到加强，全市连续7年未发生等级以上安全事故；人口计生工作全面加强，市和新干县被评为全国计生先进。此外，国防动员、民兵预备役、征兵、双拥、人民防空、国家安全工作得到加强，土地管理、审计监察、司法、统计、档案、民族宗教、对台事务、外事侨务、海关、检验检疫、工商、质监、供销、决策咨询、气象水文、新闻出版、邮政通讯等工作迈出新步伐，妇女、儿童、老龄、残疾人等事业进一步发展。

　　在过去的一年里，政府注重加强自身建设。坚持依法行政，自觉接受市人大及其常委会的法律监督、工作监督和市政协民主监督。认真规范政府重大事项决策程序，健全公众参与、专家论证咨询和政府集体决策相结合的决策机制，坚持重大事项集体研究并向市委专报请示、向市人大报告、向市政协通报。办理人大代表建议170件、政协提案212件，办结率和满意率达100%。积极推进政务信息公开，建立完善市长直通电话、市长手机、市长信箱、网上信访、信访接待、行政投诉、行风热线等平台建设和办理制度，政府工作透明度不断增强。建立健全市、县、乡、村四级行政（便民）服务体系，深化“两集中两到位”改革，精简市级行政许可项目70项，精简率为22.65%，办理时限压缩37%，深入开展“创先争优”、“创业服务年”、“三民”活动，机关工作实现提质提速提效。全年实现客商零投诉，政务和发展环境正在成为吉安招商的响亮品牌。建立工程建设领域“五位一体”监管机制，切实加强政府廉政建设和反腐败工作力度，政风建设有新进步。

　　各位代表，2010年我们为“十一五”发展划上了圆满句号。整个“十一五”时期，成为我市经济发展速度最快、城乡面貌变化最大、产业集聚成效最明显、人民生活改善最显著的时期之一。五年来，经济总量实现新跨越。实现生产总值、社会消费品零售总额、利用外资五年翻一番，财政总收入、地方财政收入三年翻一番，固定资产投资五年翻两番，规模工业增加值四年翻两番，外贸出口五年翻三番。全市财政收入占GDP比重五年提高4.2个百分点，税收占财政收入比重五年提高7.8个百分点。五年来，城镇化进程不断加快。全市新增城镇建成区面积62.35平方公里，达到190平方公里；新增城镇人口45.1万人，达到198.2万人；城镇化率提升8.9个百分点。中心城区相继跨入中国优秀旅游城市、国家园林城市、国家双拥模范城市、省级卫生城市行列。五年来，基础设施不断完善。吉井铁路、泰井及武吉高速、华能井冈山电厂二期等一批重大项目先后建成。全市新增铁路通车里程80公里、达到402公里，新增高速公路通车里程120公里、达到319公里，新建和改造国省道1200公里、农村公路8371公里，100%行政村通水泥（油）路，100%完成渡改桥任务，区域性综合交通枢纽地位正在形成；新增电力装机132万千瓦，电力总装机接近300万千瓦，居全省之首；全市固话用户53.4万户、移动用户268万户、互联网用户21万户，实现了通讯网络全覆盖。五年来，改革开放深入推进。国有工业企业改革全面完成，商贸流通、粮食、交通、农业、林业、水利、农垦、城建等非工系统改革扎实推进，医药卫生体制、投资、财税、金融、林权制度、政府机构改革步伐加快，对外开放扩大，出口总额、利用外资实现新突破。五年来，社会事业不断进步。科技创新能力增强，科技进步对经济增长的贡献率达到50%，天人集团自主创新项目获国家科技进步二等奖，是我市获得的国家级最高奖项；九年义务教育全面普及，职业教育、学前教育得到发展，井冈山大学成功升格为综合性大学；文学文艺作品屡获国家和省级大奖；公共卫生体系不断完善，城乡医疗卫生条件得到有效改善。五年来，群众生活水平不断提高。五年投入民生资金83亿元，全市城镇居民人均可支配收入和农村居民人均纯收入分别是“十五”末的1.78倍和1.66倍。城乡困难群众最低生活保障、义务教育“两免一补”、困难群众医疗救助、居民合作医疗保障制度“四个全覆盖”基本实现。新增保障性住房124万平方米，困难群众住房条件明显改善。

　　回顾五年历程，我们深切体会到，推进吉安经济社会发展，必须坚持解放思想、实事求是、与时俱进，按照科学发展观要求，不断探索符合吉安实际的发展新路，努力做大总量，加快集聚，进位赶超，跨越发展；必须坚持深化改革、扩大开放，以改革创新的办法、攻坚克难的勇气，有效破解体制机制和发展中的矛盾和问题，坚定不移实施大开放，不断提升开放型经济的能力和水平，以大开放促进大发展；必须坚持“主攻项目、决战两区”战略，以重大项目为主抓手，以园区和城区为主战场，千方百计抓项目、增投入，全力推进新型工业化和城镇化进程；必须坚持统筹城乡发展、加快县域经济发展，集中力量支持吉泰走廊率先发展，协调推进农业产业化、现代化和新农村建设；必须坚持经济建设与生态保护相结合，坚守“绿色发展”之路，在保护生态中加快发展，在加快经济发展中建设生态文明，促进人与自然和谐相处；必须坚持发展为了人民、发展依靠人民、发展成果由人民共享，做到经济发展与改善民生的有机统一；必须坚持弘扬井冈山精神，努力营造风清气正、团结和谐、埋头苦干、奋勇争先的良好氛围，始终保持“静悄悄急行军”的良好态势。这既是五年来各项工作取得突出成绩的重要保证，也是我们今后更好更快发展的宝贵经验和宝贵精神财富。

　　各位代表，吉安的变化令人鼓舞，吉安的发展振奋人心。“十一五”发展成就，是党中央、国务院重视关心的结果，是省委、省政府正确领导的结果，是市委带领全市人民团结奋斗的结果，是社会各界支持帮助的结果。我们由衷感谢市人大和人大代表的依法监督与大力支持，由衷感谢市政协和政协委员的民主监督与建言献策，由衷感谢各民主党派及各界人士的热情参与和鼎力相助，由衷感谢四海宾朋、国内外客商加盟发展，共筑辉煌。在此，我代表市人民政府，向各位人大代表、政协委员，向奋斗在各条战线的全市广大干部群众，向离退休老同志，向各民主党派、工商联、无党派人士、各人民团体，向驻吉人民解放军和武警官兵、公安干警，向中央和省驻吉单位，向所有关心、支持和参与吉安建设的海内外朋友，表示崇高的敬意和衷心的感谢！

　　在肯定成绩的同时，我们也清醒地看到，全市经济社会发展中还存在不少矛盾和问题：一是综合经济实力仍然不强，仍处在总量偏小、结构不优、人均水平较低阶段，重大项目和优势骨干企业还不多，产业集聚度还不高，消费对经济增长的贡献率偏低，经济欠发达的市情尚未根本改变，加快发展的任务还十分繁重和艰巨；二是城镇化水平仍然偏低，城市功能还不够完善，现代服务业规模小、发展慢、层次低，城区人气、商气不旺的问题依然突出，面临做大城镇规模、完善城镇功能、繁荣城市经济和集聚城市人口的多重压力；三是农村基础设施仍然薄弱，农业产业化、现代化水平和农业经济效益还不够高，农民持续增收的压力较大；四是要素制约形势仍然严峻，科技创新、创业创造能力还不强，土地供应紧张将是长期趋势，中小企业融资难仍比较突出，企业招工难不同程度地存在；五是城乡居民收入水平还不高，教育、卫生质量及管理和社会事业发展与人民群众的企盼还有差距，社会保障体系还不完善、保障水平偏低，改善民生和解决群众合理诉求的任务还很艰巨，维持物价和社会稳定的压力依然较大；六是发展环境仍然有待改善，政府行政水平、行政效能和公共服务能力还不能完全适应发展和群众的需要。对此，我们一定会高度重视，认真加以解决。

　　二、“十二五”时期的主要任务和2011年工作安排

　　“十二五”时期，是全面建设小康社会的关键时期，也是我市转变发展方式，实现进位赶超、绿色崛起可以大有作为的重要机遇期。随着国内外经济环境发生新的变化，增长模式面临深度调整，新一轮生产力布局已经开始，新一轮科技革命正在孕育新的突破，为我们推进转型发展和创新发展提供了广阔空间；随着国家促进中部崛起战略、支持革命老区发展政策深入、鄱阳湖生态经济区建设的推进，吉安的政治优势、生态优势将进一步凸现；我市正处在工业化加速期和城镇化扩张期，发展的上升通道已经打开，本身有着强劲的内在动力和较宽阔的发展空间；尤为重要的是，省委确定的“科学发展、进位赶超、绿色崛起”的宏伟目标，市委确定的“主攻项目、决战两区，做大总量、加快集聚”的战略取向，将进一步激发全市人民求发展、求富裕、求和谐的热情和干劲。历史性的机遇和任务，增强了我们奋发有为的责任感和使命感；全市人民的期盼和信任，坚定了我们加快发展的决心和信心。

　　“十二五”时期，全市经济社会发展的指导思想是：高举中国特色社会主义伟大旗帜，以邓小平理论和“三个代表”重要思想为指导，适应国内外形势新变化，顺应人民群众过上更好生活新期待，以科学发展为主题，以加快转变经济发展方式为主线，以改革开放为动力，以实现进位赶超、绿色崛起为目标，以做大总量、加快集聚为重点，以保障和改善民生为根本落脚点，积极融入鄱阳湖生态经济区建设，深入实施“主攻项目、决战两区”战略，大力优化经济结构，加快推进新型工业化、新型城镇化、农业农村现代化，着力打造区域经济特色鲜明、竞争实力不断增强、城乡面貌明显改善、各项事业全面进步、社会和谐文明、生态优美、人民幸福的新吉安。到2015年，经济总量再翻一番，主要指标总量和人均水平在全省的位次前移，全市综合经济实力迈入全省先进行列，成为全省经济实力第二方阵的排头兵，为全面建设小康社会打下坚实基础。

　　围绕上述总体要求，我们要在实际工作中切实把握好：举全市之力推动关键领域和重点区域率先发展。按照做大总量、加快集聚的要求，创新发展模式，调优经济结构，提高发展质量，形成重要竞争力，举全市之力打造“四基地一枢纽”，加快推进吉泰走廊建设，加快发展壮大县域经济。始终做到“六个坚持”，推进科学发展。坚持做大总量，推动跨越发展；坚持转变方式，推动持续发展；坚持改革开放，推动创新发展；坚持城乡统筹，推动协调发展；坚持生态建养，推动绿色发展；坚持改善民生，推动和谐发展。注重统筹兼顾、突出重点，促进全面发展。大力推进新型工业化，加快建设实力吉安；大力推进新型城镇化，加快建设宜居吉安；促进农业农村现代化，加快建设富民吉安；大力推进旅游产业化，加快建设魅力吉安；协调发展现代服务业，加快建设繁荣吉安；加强资源节约和环境保护，加快建设绿色吉安；坚持深化改革、扩大开放，加快建设活力吉安；实施科教兴市和人才强市战略，加快建设创新吉安；推动社会事业全面进步，加快建设和谐吉安；完善公共服务和社会管理体系，加快建设平安吉安。

　　2011年是实施“十二五”规划的开局之年，也是建党90周年，做好今年的经济社会发展工作具有十分重要的意义。今年全市经济社会发展的预期目标是：生产总值增长12%；规模工业增加值增长19%；财政总收入增长18%；固定资产投资增长25%；实际利用外资增长10%以上；引进市外资金增长10.5%；外贸出口总额增长13%；社会消费品零售总额增长18%；城镇居民人均可支配收入增长11%，农村居民人均纯收入增长10%；居民消费价格指数控制在4%以内，城镇登记失业率控制在4.5%以内，人口自然增长率控制在8‰左右；万元生产总值能耗、化学需氧量排放量、二氧化硫排放量、氨氮总量等指标控制在省定范围以内。围绕上述目标，重点做好以下工作：

　　（一）加快推进吉泰走廊建设，形成全市核心竞争力，为经济社会发展发挥引领作用。建设吉泰走廊应天时、秉地利、聚人和，是发展规律的遵循，更是发展的需要。要按照构建我市加快推进新型工业化、新型城镇化、城乡一体化和体制机制创新区的战略构想，把吉泰走廊打造成优势产业的聚集区、加快发展的优强区、统筹城乡的实验区、绿色崛起的带动区。

　　强化吉泰走廊规划。抓紧完成吉泰城市群规划编制和报批，加快编制吉泰走廊总体规划、功能区划、产业布局、基础设施、公共服务等专项规划。根据资源环境承载能力和发展潜力，科学划分吉泰走廊重点开发区、重要功能区，构建发展导向明确、区域优势互补、国土空间高效利用、人与自然和谐相处的区域发展新格局。协调推进走廊城乡空间布局、产业发展、社会事业、基础设施和生态建设。创新走廊以城带乡和以工促农长效机制、土地集约利用制度、户籍管理制度、行政管理体制、人才保障制度“五大体制机制”。实现人口向城镇集中、工业向园区集中、农业向适度规模经营集中、现代服务业向中心城区集中“四个集中”，推动走廊区域联动发展、互补发展、特色发展、集群发展。力争将吉泰走廊建设上升为省级发展战略，争取更多政策、项目和资金支持。

　　加快吉泰走廊产业集聚。坚持“速度领先、技术领先、规模领先、效益领先”的理念，按照“突破上游、做大中游、集聚下游、扩大应用”的原则，集中力量聚集主导产业，明确产业定位，强化产业招商，完善产业配套，加速促进传统产业高端化、新兴产业规模化、优势产业集群化。充分发挥井开区出口加工区、海铁联运口岸通关、机电产品安全检测中心、吉水电镀集控中心、专业市场等平台作用，促进资源共用、利益共享、发展共赢，努力把吉泰走廊打造成电子信息及通讯终端产业基地，新能源、新材料及节能技术产业基地，生物新医药产业基地。力争“十二五”期间，每年引进电子信息产业及相关产业资金不少于50亿元，实现吉泰走廊工业增加值占全市工业增加值的比重达到70%。

　　着力建设吉泰城市群。加快走廊内城镇布局组团、促进功能互补、协调发展，形成以中心城市为核心，以吉水、泰和为两翼，以周边镇为卫星镇的吉泰城市群即“大吉安”城市格局。加快区域内交通、电力、水利、信息、环保、邮政等基础设施一体化建设和文教科技等社会服务设施建设，启动和实施一批重大项目，提高吉泰城市群综合承载能力。实施105国道改线，建设吉安绕城公路，改扩建319国道、朱北线，形成吉泰交通大循环；规划部署引入1000KV特高压，开工建设一批220KV、110KV、35KV电网项目，提升10KV配电网供电能力，形成吉泰城市群电力支持；以重点高中、职业学校和三甲医院建设为重点，扩张区域内优质教育和卫生资源，促进公共服务一体化；加强市政设施、公用设施、商贸网点建设，加强区内对接，促进城市群协调发展。力争到“十二五”末，吉泰城市群聚集全市近50%的城镇人口。

　　（二）加速推进新型工业化，为经济社会发展提供产业支撑。以工业园区为主战场，加速产业聚集，做大工业总量，努力实现全市支柱产业和园区工业主营业务收入“两个超千亿”目标。

　　培植千亿产业。准确把握产业发展趋势，完善产业发展规划，在坚持改造提升支柱产业的同时，积极培育壮大战略性新兴产业，力争“6+1”支柱产业主营收入达到1100亿元。重点抓好电子信息、医药化工、冶金建材产业规模的拓展和产业链的延伸，提高项目集聚度、产业依存度和财税贡献度，确保冶金建材产业主营业务收入过400亿元，电子信息和医药化工产业主营收入均达到200亿元。抓好重大新兴产业特别是杰彼新能源、优特利、天人生态、三江无纺、量一光电、金一泰、大圣光纤、吉泰环保等项目建设，推动新能源、新材料、半导体照明、微生物农药、动力电池等战略性新兴产业发展，促进结构调整、产业升级。

　　壮大百亿企业。充分利用国家产业振兴、国家中小企业技术改造项目、省高新技术产业项目等扶持政策，筛选100个亿元以上工业项目列为市重点调度项目，培植主营业务收入超10亿元企业，力争通过3～5年的努力，培育1～2户主营业务收入过百亿元的大型企业。加大对井冈山卷烟厂、江西电缆、红板电子、满坤电子、航盛电子、杰克机床、先歌音响、新琪安科技、江西碱业等一批龙头骨干企业支持力度，鼓励企业通过技术创新、开拓市场、靠大联强、低成本兼并扩张等措施，不断做大做强企业规模。大力支持企业上市，出台具体扶持政策，力争年内完成1户上市、1户申报、2户辅导备案、4户股份制改造等工作。

　　打造特色园区。强力推进以井开区为龙头的园区开发建设，确保全市工业园区基础设施投入20亿元以上，建设标准厂房100万平方米。围绕园区合理布局，抓紧工业园区规划修编和扩区，进一步优化功能区划和产业布局，强化园区生产生活配套服务，提升园区承载能力；围绕完善园区功能，重点抓好井开区吉安物流口岸区建设，抓好电子专业市场以及路网、水网、气网、标准厂房等设施建设；围绕打造特色园区，实现错位发展，重点抓好深圳产业园和各县市区特色园区建设，打造一批在全省有影响的特色产业园；围绕园区集约集优发展，严格执行工业项目准入标准，切实抓好园区企业节能减排和清洁生产，抓好生态园区试点申报，切实注重园区土地集约节约利用，提高投入产出效益，推进园区转型发展，扩大工业经济总量，新增新干、永丰、安福三个超百亿园区，全市工业园区主营业务收入跨上千亿元台阶。

　　（三）加快推进新型城镇化，为经济社会发展打造强力引擎。以中心城区为核心，以县城为重点，以重点小城镇为基础，坚定不移决战城区，力争城镇化率提高2个百分点以上。

　　推进中心城区建设。抓住高铁时代到来的历史机遇，按照“突出城南新区建设、统筹滨江新区开发、实施旧城改造”的工作方向，更多融入庐陵文化元素，完善中心城区大路网及后河二期、城西片区等详细规划，推进实施90个重点城建项目，力争完成投资140亿元。启动建设新井冈山大桥，实施吉福路改扩建。进一步加强市政设施特别是社区市政建设，继续推进老住宅小区小街小巷改造，努力改善居民生活环境。进一步完善城市功能，开工建设河东自来水厂、螺子山污水处理厂一期二阶段工程、西气东输二线管道工程，完成迴龙桥排涝泵站和城南高压线迁移工程。开工建设市体育中心、市民服务中心、工人文化宫及市妇女儿童活动中心，完成庐陵文化园一期建设，启动古街区和白鹭洲书院的修缮改造、赣西堤路堤结合建设，实施以城市绿化、亮化、美化、净化、文化为内容的城市主次街道、赣江两岸街景改造工程。新建经适住房700套、廉租住房1800套、公共租赁房400套、安置房51万平方米，改造棚户区面积37.2万平方米，启动吉州、青原“城中村”改造试点。同时，围绕提高城市管理水平，完善城管体制，完善城市防灾减灾体系规划和建设，加快“数字城市”建设步伐，推进城市信息化、精细化管理；加大地下空间开发和人防工程建设力度，完善城区配套功能；加强城市无障碍设施的新建和改造，提高城市文明程度；落实“公交优先”，加密吉泰走廊公交运行网络，完善城南、城北新区公交线路；加强物业管理市场培育与监管，提升小区物业管理水平。

　　加强中小城镇建设。将中小城镇建设作为统筹城乡一体化发展的重要抓手，进一步加快推进县（市）城区道路、市政管网改造升级，加强县（市）城区供水、供电、供气等基础设施，教育、卫生、体育等公共服务设施建设，加快商贸、宾馆等服务业发展，切实提高城关镇人均道路面积、镇区绿地率和居民集中供水率，有效增强县（市）城区承载力。按照高起点、高标准的要求，突出为农服务，彰显庐陵文化特色，抓紧完成乡镇发展规划编制，推进一批重点示范镇主要道路硬化、亮化、绿化建设，推进自来水厂、农贸市场、新农民社区建设，发展农产品加工和流通业，进一步增强城镇集聚能力，努力将一批重点小城镇打造成规模集聚、特色明显和服务带动能力强的实力乡镇。

　　提升城市商贸物流水平。中心城区按照十大商贸市场体系建设规划，扩大城市经济容量，加快推进市场建设。严格准入条件，培育市场主体。以高铁站建设为龙头，以高速公路、国省道、铁路、民航为纽带，以公交、长运、商贸、物流、仓储为载体，打造城西城市综合体，打造井开区和吉州、青原3个综合物流园区，集聚商气，带旺人气。进一步规范发展房地产市场，不断优化房地产结构，满足居民的居住需求，推动房地产业健康发展，防止大起大落。积极推进户籍制度管理改革，放宽城镇落户政策，促进人口向中心城区和小城镇集中，对暂不具备落户条件的农民工，逐步解决他们在劳动就业、社会保障、住房租购、医疗保险、子女就学等方面的实际问题。

　　（四）积极发展现代农业，为经济社会发展奠定坚实基础。把发展现代农业贯穿于新农村建设的全过程，不断提高农业效益，增加农民收入，促进农村发展。

　　做大特色产业规模。稳定发展粮食生产，推广良种良法，加快农业机械化进程，努力提高粮食单产，力争粮食播面达960万亩以上，总产达76亿斤以上。稳步推进农业产业结构调整，加快畜禽、蔬菜、水产、茶叶、烟草、井冈蜜柚、高产油茶、花卉苗木等特色产业建设，形成一批规模相对集中的现代、高效、生态农业生产基地。力争全市生猪出栏、肉牛出栏、特种水产品产量稳步增长，果业、蔬菜、油菜种植面积继续增加，新增高产油茶10万亩、井冈蜜柚6万亩、花卉苗木4万亩。抓好中心城区“菜篮子”工程。

　　加速推进产业化经营。按照生产专业化、产品商品化、资源集约化、发展持续化的要求，加大农业项目招商力度，加快引进农业龙头企业，不断提高农业产业化经营水平。整合农业产业化资金，重点扶持列入全省“双十双百工程”的成长型企业，加快引进一批农产品加工企业，形成一批产业规模大、带动辐射能力强的种养型、加工型、流通型龙头企业，新增年销售收入超3亿元企业2家，超亿元企业5家，逐步把吉安打造成重要的绿色农产品生产加工基地。通过政策引导、资金扶持等措施，大力发展农民专业合作社，下决心抓好土地流转，推广“公司＋基地＋农民专业合作社＋农户”的生产经营模式，引导农民专业合作社健康发展，不断提高组织化、市场化程度。

　　加强农田水利设施建设。继续推进水利建设大会战，继续抓好水毁工程修复和病险水库除险加固，全面完成赣江及五大支流沿线重点堤防整治加固任务。开工建设峡江水利枢纽同江防护区工程和吉水县城防洪堤及其它配套工程，抓好7个小型农田水利重点县建设，推进中小流域治理工程建设，完成大中小型灌区整治和渠道清淤，恢复和新增灌溉面积4万亩。组织实施好农业综合开发高标准农田建设示范项目，建设11个万亩连片高标准农田。同时，健全完善农技推广体系，加强重大动物疫病防控、农产品质量检测、植物保护、森林防火、暴雨山洪灾害预警、综合防灾减灾体系建设，提高农业生产技术和安全保障能力。

　　扎实推进新农村建设。科学编制乡（镇）、村组建设规划，完成850个新农村建设，启动一批集镇和中心村的整治。建设农村公路1338公里，建成农村候车亭250个。加强土地管理和农村宅基地审批，重视和规范农民发展预留地管理，减少沿路建房，遏制农村违章建房。结合峡江水利枢纽移民工程，因地制宜、依山就势建设一批移民新村。加大“一村一品”建设力度，大力发展特色种养和“农家乐”休闲产业，强化农村产业支撑。切实抓好新一轮扶贫重点村建设，做好库区、深山区移民扶贫搬迁工作。

　　（五）繁荣发展现代服务业，为经济社会发展提供持续消费需求。抓住国家扩大内需特别是增加居民消费需求的机遇，大力发育培植市场体系，发展现代服务业，充分发挥服务业在优化结构、扩大消费、促进经济增长中的重要作用。

　　做旺旅游产业。策应国家红色旅游二期规划，加快国家级井冈山红色旅游标准化示范区和全市红色旅游景区景点建设。继续办好井冈山国际杜鹃花节，深化井冈山红色经典培训，强化吉安绿色生态推介宣传，提升古色文化知名度。强力推进井冈山旅游上市和申请世界文化与自然双遗产工作。积极引入市场主体，加强市中心城区旅游业态建设，做旺中心城区旅游人气。加强青原山景区建设，完善体制机制，把青原山景区打造成为中心城区的“后花园”。加快武功山金顶景区开发步伐，力争早日对外开放。依托井冈山红色旅游，打造精品旅游线路，使全市旅游景区景点串联成线，促进山上山下联动发展。推进旅游购物中心和五星级酒店建设，大力开发休闲度假型、商务会展型、保健疗养型和其它特色旅游产品，叫响做大以井冈山为龙头的红色旅游，吸引更多游客游在吉安、留在吉安，实现全市旅游总人数、总收入新突破。

　　繁荣文化产业。积极实施文化产业项目引进发展战略，争取有重大项目列入国家级、省级文化产业示范基地支持。加大文化产业招商力度，扶持天腾科技（吉安）创意文化产业园建设，发展创意、动漫等文化产业。积极推进文化体制改革，引进和整合文化资源，培育文化市场主体。发挥吉安文化艺术中心作为文化艺术殿堂作用，引进市场机制吸引有实力的文化企业开拓发展我市演艺院线和电影院线。鼓励外资进入发展新型文化业态，繁荣文化市场，引导和满足市民文化消费需求。

　　培育发展新兴服务业。把新兴服务业发展作为新的增长点，大力发展金融、保险、证券、投资、中介、信息等现代服务业。重点发展现代金融业，抓好小额贷款公司筹建，抓好融资性担保机构的规范和组建，重点解决中小企业融资难问题。积极争取招商银行、中信银行、浦发银行及全国性的证券公司等来我市设立分支机构，培育发展资本市场。积极发展培育总部经济，支持一批实力企业建设总部经济大楼，在吉安开展结算和研发。鼓励企业高管人员落户吉安。完善农村现代流通网络，加快“万村千乡”市场建设, 升级改造1000个农家店，提高农家店的生存和发展能力。继续推进农机、汽车、摩托车、家电下乡及以旧换新工作，有效拉动农村消费。

　　（六）突出项目建设，为经济社会发展注入强劲动力。围绕打造“四基地一枢纽”，推进390个重点基础设施项目、重点产业项目、民生项目的实施，优化投资结构，保持投资持续较高增长。

　　全力向上争取项目。根据国家投资方向，重点围绕交通、能源、水利设施、农村基础设施、教育卫生设施、保障性住房等民生工程、节能减排和生态环境、自主创新能力建设、战略性新兴产业发展和中央扶持老区贫困地区建设等方面，积极论证、储备、包装一批投资额大、牵动力强的重大项目。围绕新策划的重点项目，加快深度筛选和详细论证，搞好衔接，争取支持，确保一批项目尽快通过国家和省审批、核准，努力提高项目申报的速度和效率，做到成熟一个、申报一个，争取资金到位一个，使中央、省里的政策更多地转化成我市的现实生产力。

　　加快重大项目建设。加快推进昌吉赣铁路客运专线、吉安城际铁路和厦睦高速、吉莲高速、青东公路等工程建设，争取抚吉高速公路、朱北线提升改造开工建设。全力推进峡江水利枢纽、赣江石虎塘航电枢纽、井冈山水电站、吉安市风能核能及节能技术产业基地等重大项目建设，加快万安核电站、成品油管道下载库等项目的前期工作，促使全市电力总装机尽快达到700万千瓦以上。积极推进井冈山机场扩建以及咸宁至井冈山至赣州铁路、咸宁至新余至吉安铁路、衡茶吉铁路东延吉安至南城等项目的前期工作。

　　切实加强项目管理。严格招投标及标后监管，严厉打击围标、串标和违法分包、转包等行为，维护招投标市场秩序。组织开展项目管理培训，努力提高项目质量、效率、造价管控组织水平，建立政府引导、市场运作、项目法人建设机制，完善项目绩效评估制度，确保投资效益和工程质量。加强对政府投资项目的评审，提高政府资金的使用效益。做强并规范管理政府投融资平台，力争市城投公司发行第二期债券或井开区金庐陵发行债券，为项目建设提供有力的资金支持。

　　（七）继续深化改革扩大开放，为经济社会发展构筑机制体制新优势。吉安过去的发展靠的是改革开放，今后的发展更离不开改革开放。我们要继续坚定不移地推进改革开放，为经济社会发展提供体制机制保障。

　　深入推进各项改革。继续完善国有企业改革，在巩固集体林权制度改革的基础上，抓好国有林场改革试点、完善林权抵押贷款、森林保险等配套改革和农垦企业改革，做好已改制企业的社区移交、水电改造等配套改革工作。深化农村综合改革，建立和完善以农技推广为主的农业综合服务体系，在稳定和完善农村基本经营制度的基础上，积极促进土地承包经营权合理有序流转。深化医药卫生体制改革，重点抓好233所乡镇卫生院和16所公立社区卫生服务机构实施国家基本药物制度工作，实施饮食用药安全惠民工程，为群众提供满意的医疗卫生服务。

　　全面扩大对外开放。面对沿海产业加速转移的机遇，应对中西部地区承接新一轮产业转移竞争更加激烈的局面，更加扎实有力地推进对外大开放，进一步扩大开放领域，提高开放水平，强化承接载体，优化投资环境，确保年内实际引进内资200亿元，实际利用外资4.8亿美元，均增长10%。继续把“6+1”支柱产业和战略性新兴产业作为招商引资的主攻方向，加强终端制造商和品牌商的引进，注重产业配套项目的招商；继续拓展城市基础设施、农业、旅游、现代服务业等领域的招商引资，引进一批科技含量高、辐射带动能力强、提升城市综合竞争力、财税贡献大的项目；继续优化投资环境，切实帮助解决客商生产经营中的实际问题，让外来企业放心投资、安心发展。调整优化对外贸易结构，注重扩大机电产品、高新技术产品等高附加值产品出口，大力培植出口“旗舰”企业。建设好市出口加工区。加快企业“走出去”步伐，开展对外劳务输出和工程技术承包。

　　大力发展非公经济。进一步改善非公有制经济和中小企业发展环境，激发全民创业热情。鼓励扩大民间投资，消除民间资本准入障碍，促进非公经济加快发展。切实加大创业扶持力度，努力在创业培训、项目论证、行政审批、融资信贷等方面提供优质高效服务，为非公经济发展营造公平竞争、平等准入的市场环境。重点扶持发展一批具有良好市场潜力的中小企业，培养一批快速健康成长的优势企业，为经济发展注入生机与活力。

　　（八）实施科教兴市和人才强市战略，为经济社会发展提供人才保证。加快吉安发展，教育是基础，科技是前提，人才是关键。

　　扎实推进教育优先发展。抓教育就是抓民生、抓发展、抓未来、抓核心竞争力，必须继承和发扬吉安崇文重教的优良传统，再创吉安教育的辉煌。切实落实教育优先发展和均衡发展战略，加大教育投入，优化教育布局。规范发展学前教育，巩固提高义务教育，努力完善高中教育，大力发展职业教育。重点抓好市教育园区建设，实现新白鹭洲中学、吉安新一中、吉安思源学校、特殊教育等4所新学校在新区落成，各县市区全面推进教育园区建设，力争3年内每个县市区都建有教育园区，5年内每个乡镇都建成1所中心幼儿园。大力发展职业教育，建设1所高等职业院校，推进3所市属公办职业学校退城进园，支持建设5所省级示范性中等职业学校、8所省级重点建设示范性职业学校，推进校企合作、校校联合办学。支持井冈山大学发展。狠抓教育质量，建立科学的教育质量考核评价体系，加强学校内部管理，狠抓师德师风建设，狠抓校长和教师队伍建设与管理，狠抓教学招生秩序，力争通过2～3年努力，全市教育事业发展主要指标进入全省先进行列。

　　积极推进科技创新。坚持以科技创新“六个一”工程为载体，以支撑产业化发展为主线，加快构建以企业为主体、产业为依托、市场为导向、项目为抓手、产学研相结合的区域创新体系，提高集成创新和引进消化吸收再创新能力。推广先进实用技术，实施重大科技专项，力争全市高新技术产业总产值增长30%。保护知识产权，实施质量兴市和名牌战略，积极组织申报科技计划项目。深化科技管理改革，促进科技开放合作，培育科技型企业特别是高新技术企业，加强重点研发平台、特色基地和创新团队建设，加快发展优势高新技术产业，增强区域科技创新能力。

　　注重加强人才强市。着眼总量增长、结构改善、素质提高、合理使用，统筹推进各类人才队伍建设，突出抓好创新型高层次人才、重点领域专门人才、高技能人才的培养开发。坚持招才与引智、刚性与柔性引才相结合，制定年度计划，面向重点高等院校招聘一批本科以上高层次人才，面向社会招聘一批教师、医生等专业技术人才。抓好企业人才、中高级技工的培养和引进，营造留住人才的良好氛围。推进人事制度改革，打破职称评聘中论资排辈的做法，重点向业绩突出的优秀人才倾斜，优化人才成长环境。加快建设人力资源市场，促进人才资源有效配置。组织开展优秀人才评选表彰活动，激发各类人才的创造活力和创业热情。

　　（九）注重以改善民生为重点的社会建设，为经济社会发展营造和谐环境。本着尽力而为的原则，加大民生领域投入，促进基本公共服务均等化，努力增进百姓福祉。

　　大力实施民生工程。集中建设市民服务中心，方便市民办事。坚持把就业作为民生之本，多渠道开发就业岗位，加强农民工就业培训，构建和谐劳动关系，新增城镇就业5万人。扩大城乡社会保障覆盖范围，重点推进非公有制企业职工、灵活就业人员、农民工等群体参加养老保险和医疗保险工作，抓好井冈山市、永新县第二批新农保试点。探索建立城乡低保标准正常调整机制和医疗救助网上“直补”方式，保障困难群众基本生活。提高新农合保障水平，参合率稳定在93%以上，统筹基金使用率达到85%以上，确保群众受益更多。加大扶贫济困力度，提高城乡低保户补差标准、农村五保户年供养标准、城镇“三无”特困群众的月人均供养标准、特殊群体生活补助标准。加快以居家养老为基础、社区服务为依托、机构养老为补充的基本养老服务体系建设。完成农村危房改造7000户，切实为困难群众分忧解难。加大市场价格预警、监督和调控力度，确保价格总水平基本稳定。

　　大力建设生态文明。策应鄱阳湖生态经济区建设，加快发展循环经济、低碳经济，打造“绿色吉安”。全面推进森林城市创建，进一步提升高速公路等主要通道绿化水平，完成38.76万亩造林绿化“一大四小”建设任务，注重井冈山生态功能区建设，精心实施好庐陵文化生态公园、市民广场、后河二期、河东滨江新区等一批湿地公园、生态公园绿化项目，建设城镇绿色廊道，增加绿地面积，提升绿化品位。加强节能减排，改造火电、冶金等高耗能产业，依法淘汰落后产能；规范废金属加工企业发展，提升废弃资源和再生资源回收利用水平。加强环境保护工作，切实做好赣江、禾河等重要水系的保护和治理，强化水源地、城市取水口等重点地区的环境保护，开辟中心城区备用水源；大力推进以生活污水、生活垃圾和面源污染等治理为重点的农村环境综合整治工程，改善农村人居环境。

　　大力繁荣社会事业。推进市中心人民医院城南医院和社区卫生服务网络建设，抓好县级医院业务用房建设和重点乡镇中心医院建设，满足群众医疗服务需求；加强各级医疗卫生机构医德医风建设，改善就医环境，提高服务水平。加强人口计生工作，全面开展孕前优生健康检查，加大出生人口性别比整治力度，稳定低生育水平。抓好市社会福利中心和残疾人康复设施建设，推进“三院”改造。加快广电传媒中心、博物馆、民俗馆建设，加紧实施乡镇综合文化站、广播电视“村村通”、农村文化“三项活动”、农家书屋、文化信息资源共享工程等文化广电惠民工程，推动公共文化设施向乡村覆盖。组织开展社区文化和全民健身活动，积极筹办全国七城会男子篮球赛。

　　大力维护社会和谐稳定。高度重视群众来信来访，着力化解各类矛盾纠纷，坚决防止发生重大恶性事件和群体性事件。坚持不懈抓好安全生产，坚决遏制重特大安全事故的发生。强化社会治安综合治理和应急管理，探索社会管理创新，加强民兵预备役和国防后备力量建设。扎实推进和谐平安创建活动，严厉打击各种犯罪，确保人民群众生命财产安全，不断提高人民群众的安全感。

　　（十）加强政府自身建设，为经济社会发展营造良好政务环境。完成今年的各项任务，实现“十二五”美好蓝图，对政府工作提出了新的更高要求。我们将继续按照“亲民为民、创新求进、务实高效、清正廉洁”的总体目标，深入开展“创先争优”活动，切实加强政府自身建设。

　　坚持密切联系群众，在服务群众上有新作为。牢固树立群众观念，站稳群众立场，做到心里时刻装着群众、凡事想着群众、工作依靠群众、一切为了群众，多做暖民心、得民心、稳民心的实事，多做发展见实效、百姓得实惠的好事，特别是对弱势群体和困难群众多一份关爱。进一步拓宽联系群众的渠道，完善社情民意诉求通道，深入开展“三民”活动，访民情，解民忧，保民安。大力推进依法行政和政务公开，依法保障公民的知情权、监督权和参与权。

　　坚持务实创新，在推进各项工作中开创新局面。坚持解放思想、实事求是、与时俱进，大力弘扬井冈山精神，积极谋划新思路、推出新举措、开创新局面。深入推进行政管理体制改革，严格实行行政首长问责制，认真遵守政府工作规则，建立健全决策部署、检查落实、监督反馈等工作制度，形成科学高效的行政管理体制。倡导精细务实、争先创优的工作作风，坚持重大项目现场办公、重点工作领导办公推动机制，有效化解立项报批、项目用地、企业融资用工、征地拆迁等难题。深入实践一线，努力帮助基层解决困难。强化政府工作目标管理，重点抓好七大发展指标体系考核，充分调动各地开拓进取、奋勇争先的积极性。

　　坚持勤政高效，在转变工作作风上取得新成效。大力倡导求真务实之风，坚持察实情、谋实招、求实效，聚精会神谋发展，埋头苦干抓落实。增强执行的自觉性，提高落实的主动性，真正把精力集中到“想干事”上，把本领体现在“会干事”上，把目标锁定在“干成事”上，努力形成政令通、节奏快、效率高的局面。进一步理顺部门分工，合理界定权责，加强部门协作和效能问责，形成良好的政务运行机制。规范和发展各级行政服务中心，深化行政审批制度改革，促进简政放权、审批提速。认真开展“发展提升年”活动，加强行政效能监察和政务督查，全面提高行政效率和服务质量。

　　坚持清正廉洁，在加强廉政建设中塑造新形象。坚持从严治政，加强对行政权力运行的监督，自觉接受市人大的法律监督、工作监督和市政协的民主监督，坚持重大事项集体决策和报告制度，认真办理人大代表建议和政协提案；主动听取各民主党派、工商联、无党派人士和各人民团体意见，自觉接受新闻媒体和社会公众监督。认真落实政府部门党风廉政建设责任制，建立健全惩治和预防腐败体系，加强对权力运行特别是领导干部、人财物管理使用、关键岗位的监督和制约，坚决纠正损害群众利益的不正之风，严肃查处各类违规违法案件。倡导厉行节约、勤俭办事，严禁铺张浪费，把有限的资金用在加快发展上，用在改善民生上，努力打造人民满意的高效廉洁政府。

　　各位代表，回顾过去，全市人民精诚团结，克服了重重困难，创造了辉煌业绩，展现出昂扬的精神风貌，吉安从来没有像今天这样充满生机与活力。展望未来，五年的宏图已经绘就，吉安的发展站在了新的起点上，我们深感使命光荣、责任重大。让我们在省委、省政府和市委的正确领导下，团结拼搏，奋发图强，谱写出无愧于时代要求、无愧于人民期望的科学发展新篇章！努力建设美丽富强人民幸福的新吉安！
